# Feature Selection and Parameters Optimization of SVM Using Particle Swarm Optimization for Fault Classification in Power Distribution Systems

**DOI:** 10.1155/2017/4135465

**Published:** 2017-07-11

**Authors:** Ming-Yuan Cho, Thi Thom Hoang

**Affiliations:** Department of Electrical Engineering, National Kaohsiung University of Applied Sciences, Kaohsiung, Taiwan

## Abstract

Fast and accurate fault classification is essential to power system operations. In this paper, in order to classify electrical faults in radial distribution systems, a particle swarm optimization (PSO) based support vector machine (SVM) classifier has been proposed. The proposed PSO based SVM classifier is able to select appropriate input features and optimize SVM parameters to increase classification accuracy. Further, a time-domain reflectometry (TDR) method with a pseudorandom binary sequence (PRBS) stimulus has been used to generate a dataset for purposes of classification. The proposed technique has been tested on a typical radial distribution network to identify ten different types of faults considering 12 given input features generated by using Simulink software and MATLAB Toolbox. The success rate of the SVM classifier is over 97%, which demonstrates the effectiveness and high efficiency of the developed method.

## 1. Introduction

Distribution networks deliver electrical energy from transmission systems to consumers and are important and integral part of all power systems. Once an electrical fault occurs in any distribution feeder, immediate fault classification plays an important role in postfault analysis and power supply restoration. The accuracy of the fault type information assists the fault diagnosis system not only to locate the electrical faults promptly but also to ensure power quality as well as reliability of the system [1, 2].

A variety of approaches have been developed to build an effective fault classifier in electrical distribution feeders. As the amount of power delivered by a distribution system significantly increases, it is essential to focus on fault classification schemes. The studies of fault classification in distribution feeder can be divided into three separate categories, as follows: (1) impedance based method [3, 4], (2) travelling wave based method [5, 6], (3) and artificial intelligence based method [7, 8]. The most common method for fault classification in power systems is known as time-domain reflectometry (TDR) [9–11].

TDR is rather simple to implement; however, it is not a perfect fault-location method since any single pulse stimulus injected into the electrical line is quickly attenuated along that line, causing fault location and classification to become inaccurate. To overcome this problem, an improved TDR method using incident pseudorandom binary sequence (PRBS) excitation is proposed to locate such faults in [12]; however, it should be noted that it is only applied for high-power transmission lines. Actually, it is quite difficult to apply the TDR method to find faults in distribution feeders because of the various junctions and ends of branched network involved. As a result, various reflected responses may occur in the reflectometry trace [13]. Therefore, an intelligent algorithm is required to extract fault location information on a multiple-branched network from the reflectometry trace provided. SVM has been used successfully to resolve classification issues for a wide range of applications because of its strongly regularized characteristic and rapid training speed [14–16].

To build a SVM classifier, the aspect of feature subset selection plays an important role in detecting relevant variables in classification spaces. Principal component analysis (PCA) [17] and multidimensional scaling (MDS) [18] are two traditional methods applied to remove redundant variables in the original feature vectors. Authors in [19] proposed a Hadoop scheme to extract feature in parallel, in which hundreds of mappers are composed. In a recent paper [20], Ma and Niu used the firework algorithm to select input features by removing redundant influence in order to improve the icing forecasting of high voltage transmission line.

In addition to feature subset selection, the optimal set of SVM parameters also plays an important role in the distribution of samples in a given search space. Vapnik showed that the penalty parameter *C* and kernel function parameter such as gamma *γ* for the radial basis function (RBF) significantly affect the performance of SVM [21]. Various researches have been proposed to select these two parameters, but there is no general opinion for their settings [22]. The grid search method (GSM) is investigated to determine optimal parameters by attempting different values and selecting those values possessing the least amount of testing error [23]. Because of the computational complexity involved with GSM, genetic algorithm (GA) has been developed to improve classification accuracy and reduce training time by using a minimal number of features [24]. However, it takes significant amounts of calculation time due to the complex operational process, including inheritance, selection, recombination, and mutation. To overcome this relative problem, Kennedy and Eberhart proposed a population-based search technique known as particle swarm optimization (PSO) [25]. The primary advantage of the PSO based encoding technique is in its capacity to decrease trapped status in local optima and increase the classification accuracy as well as the training speed.

In this paper, a novel method based upon PSO techniques is developed to simultaneously optimize input features and SVM parameters in order to classify the fault types found in the distribution network. These fault types can be divided into ten classes, including single phase-to-ground faults (AG, BG, and CG), line-to-line faults (AB, AC, and BC), double line-to-ground faults (ABG, ACG, and BCG), and three-phase short-circuit faults (ABC). Further, this PSO-SVM classifier uses a dataset obtained from TDR analysis with PRBS excitation. Not only is the proposed PSO based encoding technique easy to use, but it also helps to significantly increase the success rate of the SVM classifier.

The remainder of this paper is constructed as follows. In Section 2, the theory of the proposed method is discussed, including TDR, SVM, and PSO.  Section 3 presents the modeling of a typical two-branched distribution feeder. The developed PSO based SVM fault diagnosis approach is given in Section 4. In Section 5, experimental simulation results and discussions are presented. Finally, a conclusion is presented in Section 6.

## 2. Basic Theory of the Proposed Method

### 2.1. Time-Domain Reflectometry

Time-domain reflectometry (TDR) is widely used for fault classification and location of faults in electrical transmission and distribution lines. TDR is based on a single pulse being injected into the given line or cable to be examined. Afterwards, some of the pulse energy is reflected back to source whenever it reaches the point of any discontinuities, such as electrical faults, tee joints, or line terminals. Since the propagation velocity is assumed to be constant, the fault distance can be measured based on the expected pulse transit time. Hence, the reflectometry trace will not only display the desired information of the fault type, but also determine the fault location.

**Figure 1:**
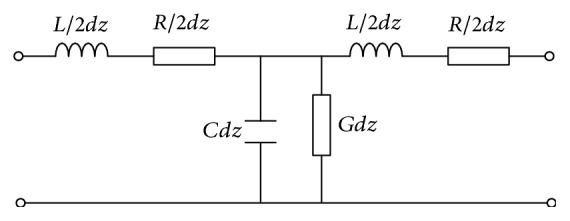
The classical model for a lumped section.

Assume a distribution line is modeled by a lumped-parameter equivalent circuit as shown in Figure 1 with a distributed series inductance *L*, resistance *R*, capacitance *C*, and conductance *G*.

A voltage introduced at the generator will require a certain amount of time to propagate along the line represented in the following equation:

(1) ∂vx,t∂x=−Rix,t−L∂ix,t∂t,∂ix,t∂x=−Gvx,t−C∂vx,t∂t,

where *v*(*x*, *t*) and *i*(*x*, *t*) are the forward travelling voltage and current waves, respectively. The amplitude of incident pulse will be attenuated along the line and the phase of the voltage travelling along the line will be distorted resulting from varying frequency [26]. The attenuation and phase shift are determined by the propagation coefficient, as shown in

(2) $$\begin{array}{c} \gamma =\sqrt{\left(R+j\omega L\right)\left(G+j\omega C\right)}=\alpha +j\beta , \end{array}$$

where *α* and *β* are the attenuation coefficient and the phase change coefficient, respectively. The velocity at which the voltage moves down the line can be defined in

(3) $$\begin{array}{c} \upsilon =\frac{\omega }{\beta }. \end{array}$$

From (1), using the Laplace transform and differential equation, we can obtain

(4) vx,t=v+t−xυ+v−t+xυ,ix,t=i+t−xυ+i−t+xυ,

where *v*^+^(*t* − *x*/*υ*) and *i*^+^(*t* − *x*/*υ*) are the forward travelling voltage and current waves, respectively; *v*^−^(*t* + *x*/*υ*) and *i*^−^(*t* + *x*/*υ*) are the backward travelling voltage and current waves, respectively. Equating the coefficients of *e*^−*sx*/*υ*^, (4) can be rewritten as

(5) Ix,s=1ZCV+se−sx/υ−V−se+sx/υ,ZC=LC,

where *Z*_*C*_ is called the characteristic impedance. When the line is terminated with any load whose impedance value is other than the characteristic impedance, a reflected wave will occur at the load and then propagate back toward the source. The voltage moving down the line in this case is given by means of

(6) $$\begin{array}{c} V\left(\;l,s\;\right)=Z_{R}\left(\;s\;\right)*I\left(\;l,s\;\right), \end{array}$$

where *Z*_*R*_ is called the load impedance. This reflected wave is related to the incident wave by representation in the following equation:

(7) V−l,s=ΓsV+se−2sτ,Γ=ZR−ZCZR+ZC,τ=lυ,

where Γ is called the receiving-end voltage reflection coefficient and *τ* is called the transit time.

TDR is quite simple to implement, but it is not a perfect technique since the use of single pulse excitation that is quickly attenuated along the line. In addition, the pulse width is one of the factors that affect the accuracy rate of the reflectometry method. TDR method, using incident pseudorandom binary sequence (PRBS) excitation can solve these problems by using cross-correlation (CCR) function between the reflected wave and incident wave given by (8) for fault diagnosis in distribution feeders:

(8) $$\begin{array}{c} C_{xy}\left(\;k\;\right)=\frac{1}{L}\overset{L}{\underset{i=1}{\sum }}x\left(\;i\;\right)y\left(\;i+k\;\right), \end{array}$$

where *C*_*xy*_ is the cross-correlation (CCR) function between the reflected wave and incident wave; *x*_*i*_ is the forward signal and *y*_*i*_ is the feedback signal.

As previously mentioned, a variety of different components exist along electrical distribution lines like transformers, capacitors, tap changers, phase splitters, and so forth so it is not easy to extract fault locations from various reflections observed in the reflectometry trace. In this study, a multilayer SVM classifier is proposed as a supporting technique for the TDR method to provide fault diagnosis in multibranch distribution networks, including single phase-to-ground faults (AG, BG, and CG), line-to-line faults (AB, AC, and BC), double line-to-ground faults (ABG, ACG, and BCG), and three-phase faults (ABC).

### 2.2. Support Vector Machine

A support vector machine (SVM) was first mentioned by Vapnik in 1995, and it has become one of the most optimal techniques for data classification. It has a solid theoretical foundation based on a combination between the structural risk minimization principle and statistical machine learning theory (SLR). The main advantages of SVM are the global optimization and high generalization ability. Further, it overcomes overfitting problems and provides sparse solutions in comparison to existing methods such as artificial neuron network (ANN) and refined genetic algorithm (RGA) in fault classification.

In standard linear classification problem, for example, one should separate the set of training data, (*x*_*i*_, *y*_*i*_), *i* = 1,2,…, *m*, *m* is the number of given observations, where *x*_*i*_ ∈ *R*_*n*_ are feature vectors and *y*_*i*_ ∈ (−1, +1) are label vectors. A binary classification problem can be posed as an optimization problem in the following way:

(9) Min: 12w22+C∑i=1mξi

(10) Subjected  to: yiw×xi+b≥1−ξi,ξi≥0,  i=1,…,m,

where *C* is the regularization parameter; *ξ*_*i*_ the penalizing relaxation variables. Equation (10) means

(11) w×ϕxi+b≥+1if  yi=+1,w×ϕxi+b≥−1if  yi=−1.

It is to be noted that the nonlinear classifier may be denoted in the input space as

(12) $$\begin{array}{c} f\left(\;x\;\right)=sign\left(\;\overset{m}{\underset{i=1}{\sum }}{\alpha_{i}}^{*}\times y_{i}\times K\left(\;x_{i},y_{i}\;\right)+b^{*}\;\right), \end{array}$$

where *f*(*x*) is the decision function and the bias *b*^*∗*^ is calculated by the Karush-Kuhn-Tucker (KKT) conditions; *K*(*x*_*i*_, *y*_*i*_) is the kernel function that produces the inner product for this feature space. In this paper, the following radial basis function (RBF) is used:

(13) $$\begin{array}{c} K\left(\;x,y\;\right)=\exp\left(\;-\gamma {\left\|\;x-y\;\right\|}^{2}\;\right), \end{array}$$

where *γ* is the kernel parameter.

To obtain optimum performance, some SVM parameters need to be select property, including the regularization parameter *C* and the kernel parameter *γ*. In this work, PSO technique is applied to optimize these two parameters accordingly.

### 2.3. Particle Swarm Optimization

Particle swarm optimization (PSO) is inspired by the social and cooperative behavior displayed by various species to fill their needs in the search space. This algorithm is guided by personal experience *Pbest*, overall experience *Gbest*, and the present movement of the particles to decide their next positions in the search space. Further, the experiences are accelerated by two factors *c*_1_ and *c*_2_, and two random numbers *r*_1_ and *r*_2_ generated between [0  1]; whereas, the present movement is multiplied by an inertia factor *w*. Mathematically, updated positions of each particle in the search space can be expressed using the two equations discussed below.

The initial population (swarm) of size *N* and dimension *D* is denoted as *X* = [*X*_1_, *X*_2_,…,*X*_*N*_]^*T*^, where *T* denotes the transpose operator. Each individual particle *X*_*P*_  (*p* = 1,2,…, *N*) is given as *X*_*P*_ = [*X*_*p*,1_, *X*_*p*,2_,…,*X*_*p*,*D*_]^*T*^. Also, the initial velocity of the population is denoted as *V* = [*V*_1_, *V*_2_, *V*_3_]^*T*^. Thus, the velocity of each particle *X*_*P*_  (*p* = 1,2,…, *N*) is given as *V*_*p*_ = [*V*_*p*,1_, *V*_*p*,2_,…, *V*_*p*,*D*_]. The index *p* varies from 1 to *N* whereas the index *q* varies from 1 to *D*.

(14) Vp,qk+1=w×Vp,qk+c1r1Pbestp,qk−Xp,qk+c2r2Gbestqk−Xp,qk,

(15) Xp,qk+1=Xp,qk+Vp,qk+1.

**Figure 2:**
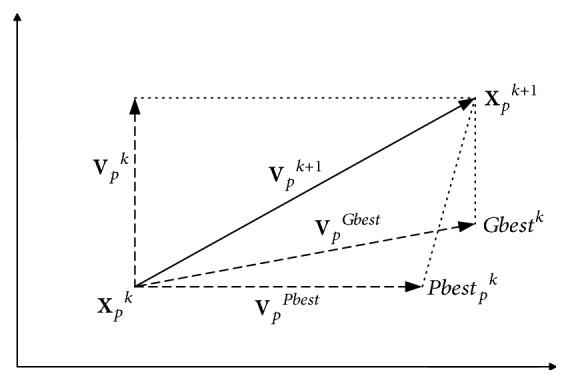
The PSO search mechanism *p*th particle at *k*th iteration.

In (14), *Pbest*_*p*,*q*_^*k*^ represents personal best *q*th component of *p*th individual, whereas *Gbest*_*q*_^*k*^ represents *q*th component of the best individual of population up to iteration *k*.  Figure 2 shows the search mechanism of PSO in a multidimensional search space.

The initial *Pbest* of each particle is their initial position, whereas the initial *Gbest* is the initial best particle position among randomly initialized population. The *Pbest* and *Gbest* of each particle are updated as follows.

At iteration *k*,

(16) If  fXpk+1fPbestpk  then  Pbestpk+1=Xpk+1  else  Pbestpk+1=PbestpkIf  fXpk+1fGbestk  then  Gbestk=Xpk+1  else  Gbestk+1=Gbestk,

where *f*(*X*) is the objective function subject to minimization. The updating procedure should be repeated until a stop condition is reached, such as a prespecified number of iterations are met. Once terminated, the *Gbest*^*k*^ and *f*(*Gbest*^*k*^) are reported as the solution of PSO technique. More details about the basic concept of PSO can be found in [27–30].

## 3. System Modeling

**Figure 3:**
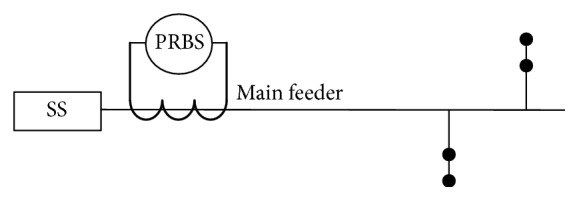
A two-branched distribution line diagram of the sample system.

An equivalent model has to be constructed by using Simulink software and MATLAB Toolbox to simulate a typical two-branched distribution feeder shown in Figure 3, in which dots represent the distribution transformers and their loads.

Two distribution transformers in the sample system are used to reduce the voltage on the distribution line to the level of customers that are distributed along a feeder. Their parameters and connection phases are shown in Table 1 [31]. It is noted that these distribution transformers are operated in a full-load condition with 0.8 lagging power factor; as a result, the sample distribution system is operated with unbalanced conditions in occurrence. The main feeder and laterals are constructed by means of overhead lines whose positive-sequence impedance is 0.131 + *j*0.364 Ω/km [31].

## 4. Developed PSO Based SVM Fault Diagnosis Approach

**Figure 4:**
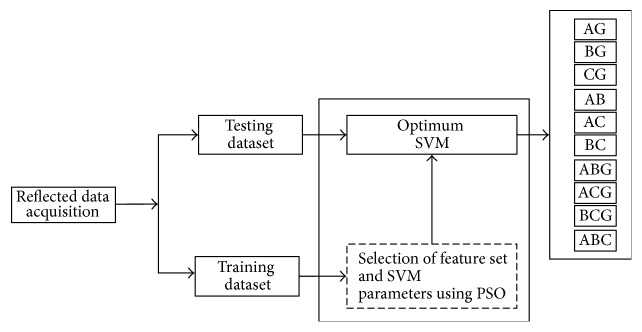
Block diagram of the proposed PSO based SVM classifier.

Since the TDR technique does not diagnose fault easily in the distribution networks hence it requires to be supported from other intelligent techniques in order to obtain the best results. This paper proposes a PSO based SVM classifier to improve the performance of the TDR method in fault classification in electrical distribution feeders. The overall structure of SVM short-circuit classifier is shown in Figure 4, in which PSO is performed to optimize the feature subset and SVM parameters. For this, the data acquisition for data preprocessing is mentioned first.

### 4.1. Data Acquisition

To obtain a suitable dataset for classification process, PRBS disturbance is injected directly into the secondary circuit of the current transformer (CT) 200/5A which is placed at the beginning of the line under test. The primary circuit of the CT is connected to the main feeder; thus the amplified PRBS is propagated along the line to diagnose any faults which may occur.

Once a fault occurs in the distribution feeder, it causes producing a reflected signal that travels between the fault location and the substation. Then, these reflected responses are cross-correlated with the incident impulse by (8) in order to reduce the impact of noise as well as surmount amplitude attenuation. It is worth noting that, for each of the fault types specified, the magnitudes of the feedback waves are different at the shortage time; as a result, the peaks of the CCR are not found to be the same. Hence, the reflected responses and CCR between the reflected wave and the incident wave are used as input feature vectors for the training phase. The total number of feature vectors is 12, and they comprise a feature vector *V* = [*v*_1_, *v*_2_,…,*v*_12_]^*T*^, in which *v*_1_–*v*_6_ are the reflected voltage and current obtained at the substation and *v*_7_–*v*_12_ are the peaks of CCR between the reflected and the incident waves.

### 4.2. Feature Extraction

For utilization of the reflectometry method, various echo responses are collected, in which some irrelevant data may be confusing to the SVM classifier and subsequently increase the training time. Feature extraction is the best effective method to select appropriate input features in order to improve the speed of training as well as to ensure the success rate of classification. For optimum feature selection in this work, PSO is employed to improve the performance of the SVM classifier. To select optimum features of the given dataset, a binary string has been optimized using PSO where each bit represents a given feature of the dataset. In the binary string, a “0” represents an ignored feature, whereas a “1” represents a selected feature of the dataset. The optimum features are those features taken from the given dataset which correspond to the optimized binary string having its bit as a “1.” For this, a given set of predefined SVM parameters has been used while the selection of features of the given dataset using PSO is made. At the end of feature selection stage, the selected strings provide the information regarding the features needed for optimizing the SVM parameters.

### 4.3. Optimum SVM Parameters

The performance of SVM is susceptible to kernel function parameter *γ* and the regularization parameter *C*, so these parameters must be carefully selected to increase the classification accuracy. In this paper, PSO technique is used to select the parameters of the SVM classifier. Performance is measured according to the classification accuracy on unseen testing data. In the learning stage, the PSO based encoding SVM model is trained based on structural risk minimization to minimize the training error. While training error improvement occurs, penalty parameter *C* and kernel function parameter *γ* are regulated by means of PSO. The regulated parameters with minimal error are reported as the most suitable parameters. As a result, the optimal parameters (*C* and *γ*) are to be obtained.

Once the optimized parameters of the SVM are obtained, then it is used for the retraining of the SVM model. After the training phase, the SVM classifier is ready to identify new samples in the testing phase. The testing set is also chosen by means of the above feature selection from the original dataset obtained by the TDR trace. Then, testing patterns are inputted to the trained multilayer SVM classifier which can identify all the 10 types of faults, including single-phase-to-ground faults (AG, BG, and CG), line-to-line faults (AB, AC, and BC), double-line-to-ground faults (ABG, ACG, and BCG), and three-phase faults (ABC).

Detailed experiment procedure for feature extraction and SVM parameter selection using PSO algorithm can be expressed using the following steps:

1. Read complete data and set *w*, *c*_1_, and *c*_2_ parameters.
2. Initialize positions **X** and velocities **V** of each particle of population.
3. Initialize sets of SVM parameters within its ranges as particle position and velocity.
4. Form SVM using training dataset and initialized positions of each particle.
5. Evaluate fitness of each particle *F*_*p*_^*k*^ = *f*(**X**_*p*_^*k*^), ∀*p*, and find the best particle index *b*.
6. Select *Pbest*_*p*_^*k*^ = **X**_*p*_^*k*^, and *Gbest*^*k*^ = **X**_*b*_^*k*^.
7. Set iteration count *k* = 1.
8. *w* = *w* max − (*w* max − *w* min) × ite/max⁡ite.
9. Update velocity and position of each particle using (14) and (15).
10. Evaluate updated fitness of each particle *F*_*p*_^*k*+1^ = *f*(**X**_*p*_^*k*+1^), ∀*p*, and find the best particle index *b*_1_.
11. Update *Pbest* of each particle ∀*p*
12. If *F*_*p*_^*k*+1^ < *F*_*p*_^*k*^ then *Pbest*_*p*_^*k*+1^ = *X*_*p*_^*k*+1^; else *Pbest*_*p*_^*k*+1^ = *Pbest*_*p*_^*k*^.
13. Update *Gbest* of population
14. If *F*_*b*1_^*k*+1^ < *F*_*b*_^*k*^ then *Gbest*^*k*+1^ < *Pbest*_*b*1_^*k*+1^ and set *b* = *b*_1_; else *Gbest*^*k*+1^ < *Gbest*^*k*^.
15. If *k*max⁡ite then *k* = *k* + 1 and go to step (6); else go to step (14).
16. Optimum solution obtained: print the results of optimum generation as *Gbest*^*k*^
17. Retrain SVM with optimum features and parameters; then identify unknown samples on testing dataset.

**Figure 5:**
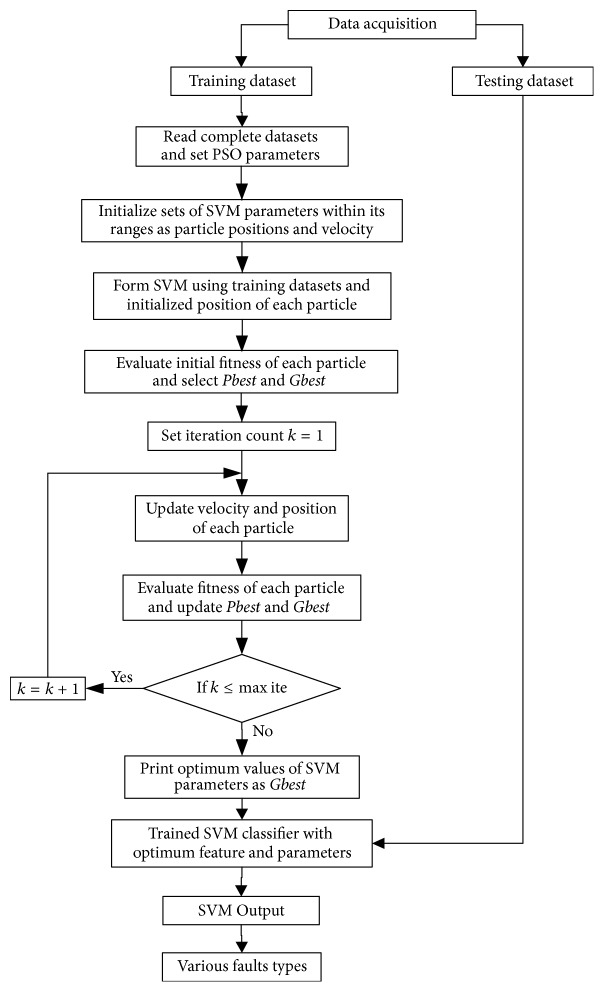
Flowchart of the proposed approach.

The experiment procedure can be visualized in Figure 5.

## 5. Test Results and Discussion

In this paper, the fault types are considered by using a 127-bit PRBS stimulus with frequency *f* = 1 MHz and a velocity of 198,000 km/s propagated along the sample system given in Figure 2. The dataset used in this study was obtained at the substation end by TDR analysis, with the number of features being 12, in which six features are considered to be the magnitudes of reflected signals and six remaining features are extracted from the peaks of CCR between the feedback wave and the forward wave. This dataset is comprised of 5700 samples generated by creating each type of fault at different locations on two laterals with varying fault impedance value. Note that training and test sets are randomly divided from the original dataset, in which 4500 and 1200 are used for training and testing set, respectively.  Table 2 only gives a few portions of the dataset for purposes of brevity, which were created by a simulation of the ten types of short-circuit fault on the first lateral, located at distances of 3 km and 4 km from the substation.

In this paper, PSO technique is used to select the features and parameters of the SVM classifier. Preliminary experiments also permit this study set population size as 10; inertia weight has been taken into account as between 0.1 and 0.5 (considered randomly at each iteration); and acceleration factors (*c*_1_ and *c*_2_) have been taken as equal to 2 with a maximum iteration set to 1000.

Table 3 gives the results of the classification accuracy for the SVM algorithm using a dataset both with and without PSO optimization. The optimum values of *C* and *γ* of SVM classifier are 181.0193 and 1.1212 without consideration of PSO and are 15.0381 and 0.0334 with consideration of PSO. From this table, it is observed that the classification accuracy in the case of using the entire feature is 93%, whereas the classification accuracy in the case of using a PSO based encoding technique is found to be 97.15%. This demonstrates the optimal efficiency of the proposed method in which PSO optimization is applied. All 12 features are autoselected from the corresponding input, and the testing success rate has been improved significantly. The remaining features are 8, which are 1–7 and 9. Furthermore, Table 3 provides the computational times for training SVM classifier. The overall simulation time taken by the SVM classifier without PSO is 134.8 seconds, whereas with PSO it is 83.54 seconds. It should be concluded that the PSO technique takes a relatively shorter computational time for training.

**Figure 6:**
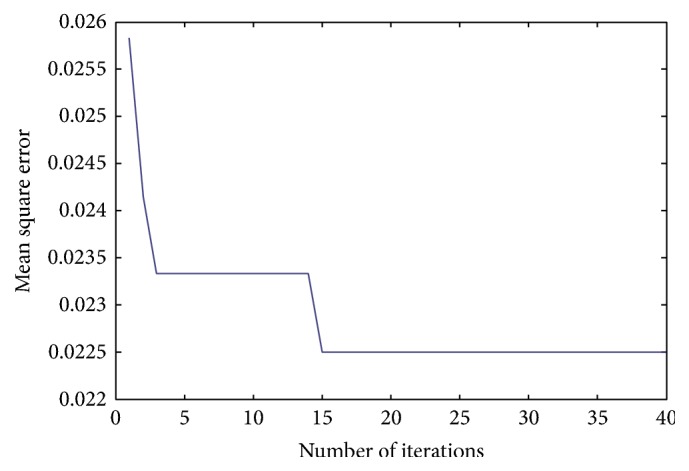
Convergence characteristic of the proposed PSO.

The convergence characteristic of the proposed PSO is shown in Figure 6. From this figure, it is can be observed that MSE beyond 15 iterations is nondecreasing; thus the optimized SVM parameters can be obtained prior to the total training time taken (83.54 sec).

## 6. Conclusions

In this paper, a multilayer support vector machine (SVM) based on optimum parameters optimization and feature selection approach has been developed to classify ten types of faults in radial distribution feeders. Particle swarm optimization (PSO) has been used as an optimizer to improve the performance of SVM classifier by selecting an appropriate feature subset and kernel parameters. Further, time-domain reflectometry (TDR) with pseudorandom binary sequence (PRBS) stimulus has been utilized for generating a fault dataset. In the proposed technique, not only does using PRBS injection overcome the stimulus distortion problem, but it also surmounts the impact of noise to provide a reliable dataset for SVM classifier. The proposed PSO based SVM classifier has been successfully applied to identify all ten types of short-circuit faults in the radial distribution network observed. The achieved high accuracy rate in classifying fault types (over 97%) demonstrates greater effectiveness over existing fault identifiers.

## Figures and Tables

**Table 1:** Parameters and connection phases of distribution transformers in the sample system.

Number	Windings connection	Phases	Secondary voltages (V)	Capacity (kVA)	Impedance (*Z*%)
1	Delta-Wye-Gnd.	A, B, C	220	500	1.89
2	Delta-Delta	A, B, C	220	500	1.89

**Table 2:** Dataset of ten fault types located at distances of 3 km and 4 km from the substation.

	*v*_a_	*v*_b_	*v*_c_	*i*_a_	*i*_b_	*i*_c_	cc-*v*_a_	cc-*v*_b_	cc-*v*_c_	cc-*i*_a_	cc-*i*_b_	cc-*i*_c_
AG	1.9197	−0.3071	0.1245	4.9815	−0.7968	0.3232	0.5941	−0.0950	0.0385	0.0502	−0.0080	0.0033
	0.6990	−0.1118	0.0453	1.5998	−0.2559	0.1038	3.5687	−0.5708	0.2315	3.0765	−0.4921	0.1996
BG	1.4521	0.7277	0.5122	3.7681	1.8884	1.3290	0.4494	0.2252	0.1585	0.0380	0.0190	0.0134
	0.5287	0.2650	0.1865	1.2101	0.6064	0.4268	2.6995	1.3528	0.9521	2.3271	1.1662	0.8208
CG	0.4648	4.5783	3.1718	0.0857	0.8445	0.5851	0.0275	0.2711	0.1878	0.0237	0.2331	0.1615
	0.0880	0.8668	0.6005	0.2284	2.2492	1.5582	0.0272	0.2683	0.1858	0.0023	0.0227	0.0157
BCG	−8.2016	9.6684	16.2648	−2.5267	2.9785	5.0107	−0.1137	0.1340	0.2254	−0.1137	0.1340	0.2254
	−4.1309	4.8697	8.1921	−0.7620	0.8983	1.5112	−0.2446	0.2884	0.4852	−0.2104	0.2480	0.4172
ACG	−1.2835	2.5576	4.8025	−0.9796	1.9519	3.6650	−1.6907	3.3688	6.3257	−1.4240	2.8375	5.3279
	−1.4241	1.7834	3.8278	−1.0868	1.3610	2.9212	−1.8757	2.3491	5.0419	−1.5799	1.9786	4.2466
ABG	−1.1327	0.0679	2.6912	−2.9393	0.1763	6.9832	−0.3506	0.0210	0.8329	−0.0296	0.0018	0.0704
	−2.0970	0.1258	4.9821	−1.6003	0.0960	3.8021	−2.7621	0.1657	6.5623	−2.3265	0.1395	5.5272
AB	−7.4589	−4.8688	17.7206	−1.3759	−0.8981	3.2688	−0.4417	−0.2883	1.0495	−0.3798	−0.2479	0.9024
	−1.4121	−0.9218	3.3549	−3.6643	−2.3918	8.7055	−0.4370	−0.2853	1.0383	−0.0369	−0.0241	0.0877
AC	−1.0143	−1.2113	7.8915	−0.7741	−0.9244	6.0225	−1.3360	−1.5955	10.3945	−1.1253	−1.3439	8.7550
	−1.5121	−7.9329	40.5259	−0.4658	−2.4439	12.4847	−0.0210	−0.1099	0.5616	−0.0210	−0.1099	0.5616
BC	2.0444	−4.2356	23.5915	0.3771	−0.7813	4.3518	0.1211	−0.2508	1.3972	0.1041	−0.2157	1.2013
	0.1409	−0.2920	1.6262	0.3225	−0.6682	3.7220	0.7195	−1.4907	8.3028	0.6203	−1.2851	7.1576
ABC	0.3508	−0.3674	1.9940	0.8029	−0.8408	4.5638	1.7911	−1.8757	10.1807	1.5440	−1.6170	8.7765
	1.7837	−1.8679	10.1386	1.3612	−1.4255	7.7374	2.3494	−2.4604	13.3543	1.9788	−2.0723	11.2480

AG, BG, and CG are single phase-to-ground faults; BCG, ACG, and ABG are double line-to-ground faults; AB, AC, and BC are line-to-line faults; ABC is three-phase faults; *v*_a_, *v*_b_, *v*_c_, *i*_a_, *i*_b_, and *i*_c_ are magnitudes of reflected voltages and currents, respectively; cc-*v*_a_, cc-*v*_b_, cc-*v*_c_, cc-*i*_a_, cc-*i*_b_, and cc-*i*_c_ are CCR between reflected signal and incident signal.

**Table 3:** Results of SVM classification without and with considering PSO optimization techniques.

SVM classifier	Number of features	*C*	*γ*	Classification accuracy (%)	Training time (s)
Without PSO	12	181.0193	1.1212	93.00	134.8
With PSO	8	15.0381	0.0334	97.15	83.54
